# Oxidative resistance of leukemic stem cells and oxidative damage to hematopoietic stem cells under pro-oxidative therapy

**DOI:** 10.1038/s41419-020-2488-y

**Published:** 2020-04-27

**Authors:** Yongfeng Chen, Yong Liang, Xingjing Luo, Qiongying Hu

**Affiliations:** Department of Basic Medical Sciences, Medical College of Taizhou University, Taizhou 318000 Zhejiang, China

**Keywords:** Haematological diseases, Pathogenesis

## Abstract

Leukemic stem cells (LSCs) and hematopoietic stem cells (HSCs) are both dependent on the hypoxic bone marrow (BM) microenvironment (also known as the BM niche). There is always fierce competition between the two types of cells, and the former exhibits a greater competitive advantage than the latter via multiple mechanisms. Under hypoxia, the dynamic balance between the generation and clearing of intracellular reactive oxygen species (ROS) is conducive to maintaining a quiescent state of cells. Quiescent LSCs can reside well in the BM niche, avoiding attack by chemotherapeutic agents, which is the cause of chemotherapeutic resistance and relapse in leukemia. HSCs acquire energy mainly through anaerobic glycolysis, whereas LSCs achieve energy metabolism largely through mitochondrial oxidative respiration. Mitochondria are the primary site of ROS generation. Thus, in theory, mitochondria-mediated respiration will cause an increase in ROS generation in LSCs and a higher intracellular oxidative stress level. The sensitivity of the cells to pro-oxidant drugs increases as well, which allows for the selective clearing of LSCs by pro-oxidative therapy. However, HSCs are also highly sensitive to changes in ROS levels, and the toxic effects of pro-oxidant drugs on HSCs poses a major challenge to pro-oxidative therapy in leukemia. Given the above facts, we reviewed studies on the oxidative resistance of LSCs and the oxidative damage to HSCs under pro-oxidative therapy. An in-depth investigation into the oxidative stress status and regulatory mechanisms of LSCs and HSCs in hypoxic environments will promote our understanding of the survival strategy employed by LSCs and the mechanism of the oxidative damage to HSCs in the BM niche, thus facilitating individualized treatment of leukemia patients and helping eliminate LSCs without disturbing normal hematopoietic cells.

## Facts

1. Redox homeostasis is vital for maintaining the quiescence of LSCs. Quiescent LSCs can reside in the BM niche to avoid attack by chemotherapeutic agents, which is the cause of chemotherapeutic resistance and relapse in leukemia.
2. Theoretically speaking, LSCs undergoing mitochondria-mediated respiration will also exhibit an increased sensitivity to pro-oxidant drugs, which provides a basis for the leukemia treatments targeting redox homeostasis. However, the BM niche can protect LSCs from pro-oxidative treatments, and LSCs can also resist oxidative damage through antioxidative mechanisms.
3. For HSCs in hypoxic BM niches, a low ROS level is conducive to maintaining their stem cell features. A higher ROS level not only disrupts the quiescent state of HSCs but also may kill bone marrow hematopoietic stem cells (BMHSCs) or even cause BM suppression. It is then necessary to reduce the dosage or even completely stop chemotherapy.

## Open questions

1. In pro-oxidative treatment of leukemia, how can the protective effects of the BM niche on LSCs be blocked?
2. Leukemia is a disease of high heterogeneity, and the oxidative stress of leukemic cells varies across patients and dynamically within the same patient. A level of ROS that is too low during pro-oxidative treatment may be beneficial for the survival and proliferation of leukemic cells. However, if it is too high, it will exacerbate the damage to normal cells. Therefore, it is necessary to determine the optimal pro-oxidative treatment.
3. At present, studies on pro-oxidant therapy for leukemia are mainly conducted in animal experiments or in vitro cell experiments. Therefore, they cannot fully reflect the real situations in vivo. More convincing evidence is needed to reveal what the true redox state of LSCs and HSCs is in different types and stages of leukemia and whether there are significant differences in the sensitivity of the two cells to ROS.
4. The regulatory mechanism for redox homeostasis may differ between LSCs and HSCs. As such, is it possible to identify specific targets for pro-oxidative treatment to kill LSCs while avoiding damage to BMHSCs?

## Introduction

Leukemia is a hematopoietic malignancy caused by mutations in BMHSCs or hematopoietic progenitor cells (HPCs). With the application of novel chemotherapeutic drugs and the progress in hematopoietic stem cells (HSCs) transplantation, the remission rate and disease-free survival of leukemia patients have improved. However, during chemotherapy, leukemic stem cells (LSCs) may reside inside the BM niche in a quiescent state, evading the killing power of the chemotherapeutic agents. Thus, the protective effect of the BM niche on residual LSCs is the cause of chemotherapeutic resistance and relapse in leukemia^1,2^.

In a hypoxic BM niche, maintenance of quiescence and the biological functions of HSCs and LSCs, cell survival, and proliferation are closely related to the intracellular reactive oxygen species (ROS) level and oxidative stress status^3^. Much evidence in recent years has indicated that targeting the BM niche and disrupting redox homeostasis may be a new treatment strategy for leukemia^4^. However, HSCs are also highly sensitive to an increased ROS level. How to reduce the cytotoxic effects of ROS on HSCs while killing LSCs with a high ROS level represents another challenge in pro-oxidant therapy for leukemia. Therefore, an in-depth investigation into the oxidative stress status and regulatory mechanisms of HSCs and LSCs in hypoxic environments will promote our understanding of the survival strategy of LSCs in the BM niche and the limitations of HSCs in resisting oxidative injury. This understanding will help to develop individualized treatments that can protect normal BMHSCs while eradicating LSCs.

### Hypoxia is significant for maintaining the biological functions of HSCs

HSCs in a hypoxic BM microenvironment mainly rely on anaerobic glycolysis for energy, and the ROS level associated with anaerobic glycolysis is relatively low. However, the differentiation of HSCs may lead to dynamic changes in the ROS level, and it was found that low endogenous ROS levels were crucial for maintaining the quiescence of HSCs^5^. Whereas an excessively high ROS level will drive HSCs to shift from the quiescent state, and their self-renewal capacity will be reduced, causing oxidative injury or even death of HSCs. Moreover, an increased production of ROS has also been associated with genomic instability and enhanced DNA damage, including double-strand breaks, and performs a signaling function to promote cell proliferation and migration, thus contributing to leukemic cell transformation^6,7^. It should be noted that ROS also has a pivotal role in innate immunity by acting as signaling molecules and as a direct effector that kills pathogens via phagocytosis. However, a persistently low ROS level in HSCs will not only lead to loss of stem cell function but also cause opportunistic infections^8^. Thus, the balance of ROS levels is critical for maintaining the biological functions of HSCs and host immunity.

**Fig. 1:**
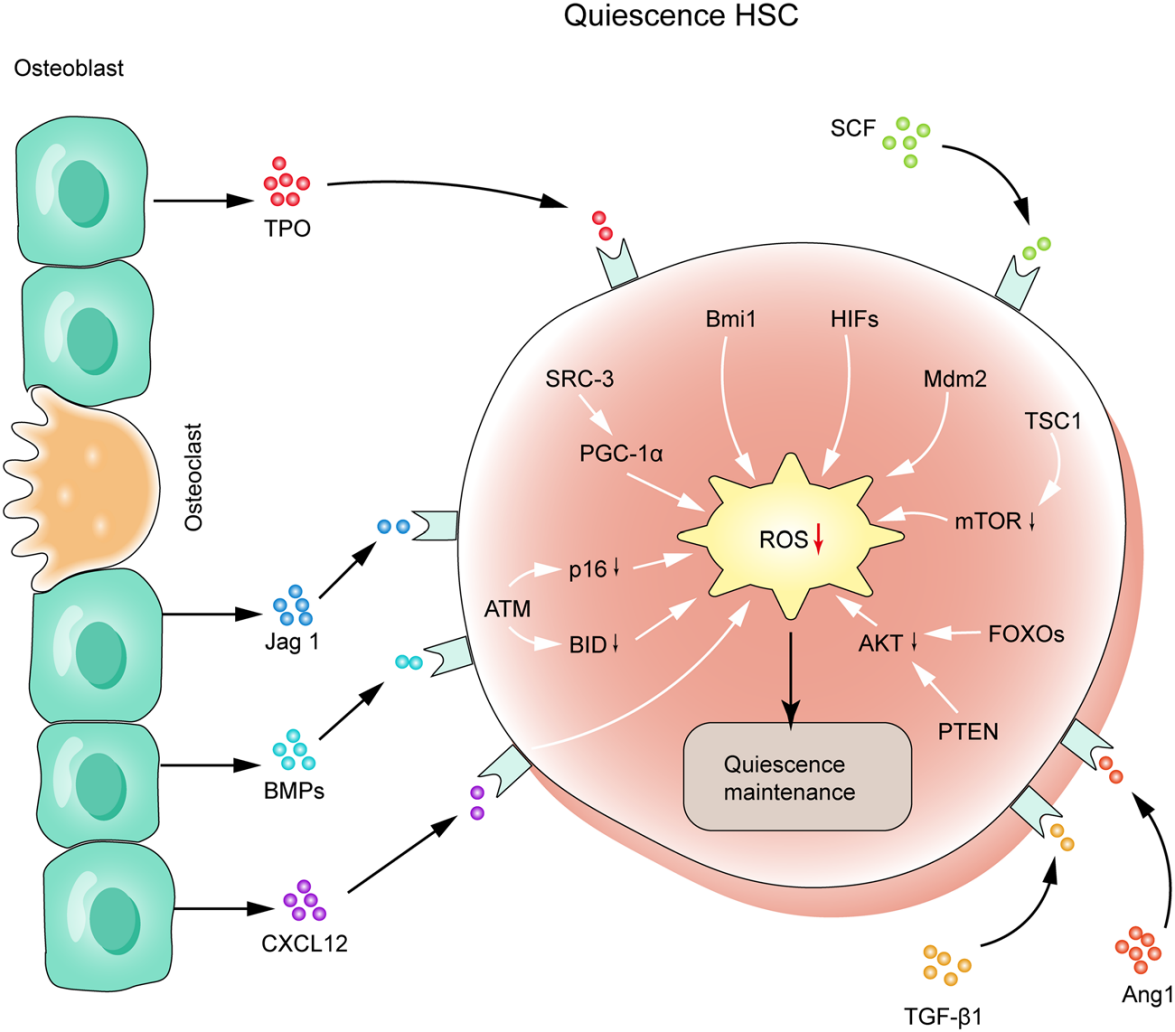
Maintenance of redox homeostasis and quiescence of HSCs in the BM niche. The ROS level of HSCs in quiescence is regulated by a complex signaling network consisting of ATM, HIFs, FoxOs, SRC3, etc., which work together to maintain a low intracellular ROS level. The interaction between HSCs and BMSCs in the BM niche has a vital role in the long-term stability of HSCs. TPO, SCF, *TGF-β1*, and BMPs produced by BMSCs are all important regulators of the quiescence of HSCs.

In terms of the metabolism of HSCs, many regulatory mechanisms, including high antioxidant defense and a diversity of regulatory molecules, such as P53, FOXO3, Akt, MAPK, hypoxia inducible factor 1 (HIF-1) and ataxia telangiectasia mutated (ATM), are involved in maintaining the low ROS level of HSCs to protect HSCs from oxidative stress-induced injury^9–13^. Bone marrow stromal cells (BMSCs) residing in the HSC niche also has a vital role in maintaining the redox homeostasis of HSCs, and ROS in HSCs can be transferred to BMSCs, maintaining a low ROS level^14^. In addition, stromal cells can regulate the quiescence, proliferation, and differentiation of HSCs through direct contact with HSCs and secretion of a variety of cytokines via multiple signaling pathways (Fig. 1). Among them, the chemokine ligand 12 (CXCL12)/C-X-C chemokine receptor type 4 (CXCR4) axis has the most important role in the interaction between BMSCs and HSCs^15^. In addition, different types of immune cells and nerve cells are also involved in the regulation of the HSC microenvironment^16,17^.

### LSCs are more adapted to the hypoxic environment of the BM niche than HSCs and inhibit the latter

LSCs and HSCs share similar self-renewal and differentiation features and other biological functions. For this reason, LSCs and HSCs are engaged in intense competition in the BM microenvironment, and the former usually have more advantages^18,19^. It has been shown that a hypoxic BM microenvironment can promote the synthesis of HIF-1a, which further induces the upregulation of CXCR4 on the surface of LSCs. The migration ability of LSCs can be enhanced through the interaction between CXCL12 and CXCR4, which facilitates the anchoring of leukemic cells in the BM niche and their quiescence. With the LSCs better protected in the BM niche than they are in circulation, their resistance to chemotherapeutic agents increases as well^20–22^.

**Fig. 2:**
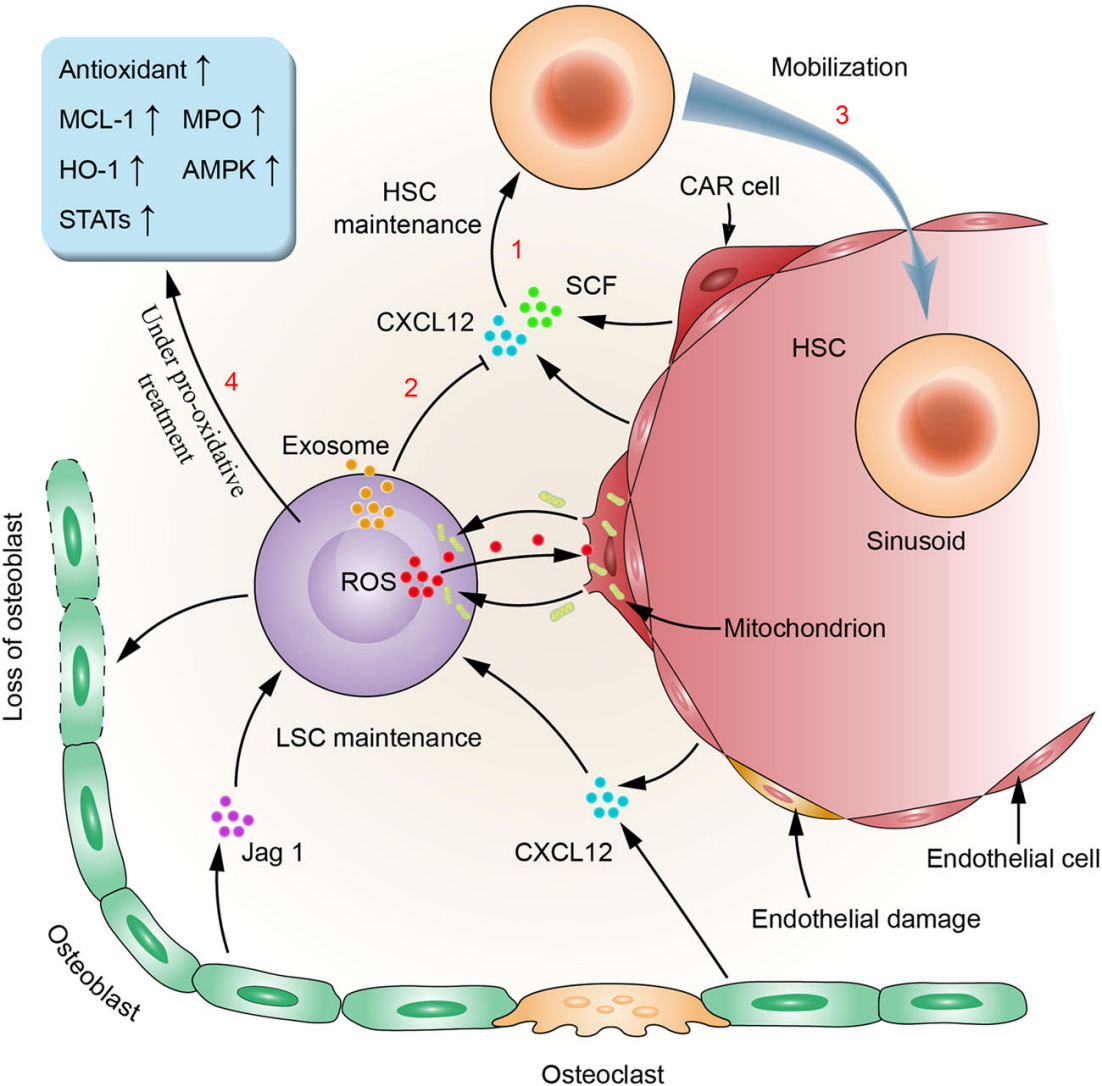
LSCs’ remodeling of the BM niche, inhibition of HSCs, and resistance against oxidative stress. LSCs have a remodeling effect of the BM niche by multiple pathways, such as activating the production of abnormal osteoblastic lineage cells from mesenchymal progenitor cells (MPCs). The interaction between LSCs and MSCs, including the transfer of ROS and mitochondria, is conducive to maintaining a low intracellular ROS level and energy metabolism of LSCs under a hypoxic environment. In addition, it has been indicated that 1. hematopoietic stem cell-supporting and retention factors secreted by bone marrow matrix cells, such as SCF and CXCL12 etc., have an important role in HSCs maintenance; 2. exosomes derived from leukemic cells may interfere and destroy HSCs maintenance by downregulating SCF and CXCL12.; 3 HSCs mobilization in bone marrow niche is accelerated; 4. In the pro-oxidative treatment, LSCs can respond by upregulating antioxidant, MCL-1, MPO, and HO-1. As leukemia is a highly heterogeneous disease, the survival and redox regulation mechanisms of LSCs in the BM niche may vary for different types of leukemia. More studies are needed for revelation in this subject.

As leukemic cells proliferate massively in the BM niche and secondary anemia occurs, hypoxia within the niche is exacerbated. Compared with normal HSCs, leukemic cells are more resistant to hypoxia. Goto et al.^23^ showed that leukemic cells can better survive in a hypoxic environment than HSCs by reducing ROS generation and enhancing ROS clearance. The leukemic cells can also release exosomes containing a variety of microRNAs, such as miR-210, which are then transported to the endothelial cells to inhibit the expression of the antiangiogenic factor EPH-related receptor tyrosine kinase ligand 3 (EFNA3), thus promoting angiogenesis^24^. In addition, HIF-1a^25–28^ and many cytokines, including granulocyte colony-stimulating factor (G-CSF), granulocyte-macrophage colony-stimulating factor, CXCL12 and angiopoietin 1 (Ang1) secreted by BMSCs, are also involved in the regulation of angiogenesis^29^. The formation of more new vessels is conducive to the tolerance of the leukemic cells to the hypoxic environment and to the supply of oxygen and nutrients to LSCs for rapid growth. Recent reports indicate that HSCs acquire energy mainly through anaerobic glycolysis, whereas LSCs maintain energy metabolism and survival largely through mitochondrial oxidative respiration^30,31^. According to recent studies, in human acute myeloid leukemia (AML) cells, the mitochondria of BMSCs can be transferred to AML cells via AML-derived tunneling nanotubes, a process that is dependent on the ROS generation mediated by nicotinamide adenine dinucleotide phosphate (NADPH) oxidases (NOX)-dependent oxidative stress. Thus, more energy is supplied to AML cells through mitochondrial oxidative phosphorylation (Fig. 2). However, this phenomenon is not observed in HSCs^32^. Therefore, inhibiting angiogenesis and blocking the mitochondrial respiratory pathway of leukemic cells may help inhibit LSCs.

In addition to adaptation to the environment and competition for resources, leukemic cells can inhibit the number and activity of normal HSCs directly or by causing deterioration of the hematopoietic microenvironment. Kumar et al. showed that the expression of Dickkopf-related protein 1, which is a suppressor of normal osteogenesis and hematopoiesis, was elicited by AML-derived exosomes. Moreover, hematopoietic stem cell-supporting factors were downregulated in BM stromal cells, and their effect in supporting normal hematopoiesis was also decreased^33^. Huan et al. reported that critical retention factors (stem cell factor and CXCL12) were downregulated by AML-derived exosomes in stromal cells, and hematopoietic stem and progenitor cells were mobilized from the BM^34^. In addition, leukemic cells can induce remodeling of the BM niche by promoting the production of abnormal osteoblastic lineage cells from mesenchymal progenitor cell, thus transforming it into a normal hematopoiesis-suppressive and leukemia growth-permissive leukemia niche^35–38^.

### ROS levels are associated with the status of leukemic cells

It has long been known that increased production of ROS is a feature of tumor cells, and leukemic cells are not an exception, as they also exhibit elevated level of ROS. This feature is found in many leukemic cell lines and the cells of patients with various types of leukemia^10^. Compared with the levels in differentiated cancer cells, ROS levels are lower in cancer stem cells (CSCs), which is crucial for the survival of CSCs and the maintenance of their stemness. In contrast, an excessively high ROS level may trigger the death of CSCs^39^.

Recent studies have shown that similar to that of CSCs, the self-renewal ability of LSCs is also closely related to the ROS level and oxidative stress of the cells. According to Herault et al.^40^, LSCs highly expressed glutathione peroxidase 3, and their ROS level was low, thereby maintaining the properties of stem cells. Lagadinou et al. analyzed ROS generation in primary AML cells, and it was found that AML cells with low ROS were more primitive than those with high ROS, exhibiting an immunophenotype and functional features of LSCs and largely being G_0_-stage, quiescent cells. This relatively dormant condition likely enables LSCs to persist, even under restrictive conditions such as low nutrient or oxygen levels^31^. LSCs account for ~0.1–1.0% of leukemic cells in leukemia patients. Quiescent LSCs can reside in the BM niche to avoid attack by chemotherapeutic agents, which is the cause of chemotherapeutic resistance and relapse in leukemia. It has been shown that direct contact with BMSCs and regulation of the signaling molecules angiopoietin 1 (Ang1) and B-cell lymphoma 2 (Bcl-2) has important roles in maintaining the quiescence of LSCs^31,41,42^. Disrupting the quiescence of LSCs and inducing cell entry into the cell cycle in combination with administration of cell cycle-dependent chemotherapeutic agents can help eradicate the residual LSCs in clinical treatments for leukemia.

Compared with the level in more primitive leukemic cells in a quiescent state, the ROS level is higher in leukemic cells that are proliferating more^30^. Previous studies have shown that the mechanism of ROS generation in leukemic cells is very complex. When leukemic cells acquire energy through mitochondrial respiration, the mitochondrial respiratory chain become a very important source of intracellular ROS^30^. According to the literature, among various types of leukemia cells, including AML, chronic myeloid leukemia (CML) and promyelocytic leukemia cells, an increase in NOX activity is observed, indicating that NOX’s constitutive activation is a very important source of intracellular ROS for LSCs^43–45^. Moreover, activated FMS-like tyrosine kinase and oncogenes such as BCR/ABL, cellular-myelocytomatosis viral oncogene and Ras are all closely related to changes in redox homeostasis in leukemic cells and increased ROS levels^9,46,47^. It has been found that antioxidant defense is decreased in different types of leukemia^48–51^, indicating that an imbalance between the oxidative and antioxidative systems may be one of the reasons for increased ROS levels in leukemic cells.

Recently, Bourgeais et al.^52^ found that the persistent activation of signal transducer and activator of transcription (STAT)5 induced by BCR-ABL promoted ROS production in CML cells by inhibiting catalase (CAT) and glutaredoxin-1 (Glrx1) expression; however, when leukemic cells were cocultured with BM stromal cells to mimic a leukemic niche, CAT and Glrx1 were upregulated, causing downregulation of ROS levels and enhancement of leukemic cell quiescence. Given the facts above, antioxidant capacity may be related to the status of leukemia cells. It is believed that the downmodulation of some antioxidant systems contributes to the high level of ROS found in leukemic cells and that the upregulation of antioxidants allows the cells to survive under permanent oxidative stress without surpassing a deadly threshold. Furthermore, antioxidant upregulation promotes intracellular ROS elimination, maintaining cellular quiescence^10^.

### Targeting ROS in treatment for leukemia

Mitochondria are the primary site of ROS generation. Thus, in theory, mitochondria-mediated respiration will cause an increase in ROS generation in leukemia cells and a higher intracellular oxidative stress level. The sensitivity of the cells to pro-oxidative drugs increases as well, which allows for the selective clearing of LSCs by pro-oxidative therapy^53–57^. The application of pro-oxidant chemotherapeutic agents may cause death of leukemia cells by increasing ROS, protein oxidation and mutation, lipid peroxidation, and mitochondrial stress and activating the G_2_/M phase cell cycle checkpoint^10,58^.

Studies have shown that a variety of chemotherapeutic agents for leukemia, including vincristine, doxorubicin, and cytosine arabinoside, work by promoting ROS generation^59–62^. Constant optimization and the combined use of chemotherapeutic agents can help improve the outcomes of pro-oxidative treatment for leukemia^63^. Mitochondria are the main site for intracellular ROS generation. Therefore, targeting mitochondria is a reasonable strategy to disrupt the redox balance of the cells, induce oxidative stress and promote the apoptosis of leukemia cells^64^. A variety of mitochondrial inhibitors that can promote ROS generation are undergoing clinical trials for their role in leukemia treatment. Metformin, an antidiabetic drug, has been proven to be capable of inhibiting mitochondrial ATP generation and increasing ROS levels^65^. Adaphostine is another drug with proven ability to increase ROS levels by inhibiting mitochondrial respiration, this drug can overcome the resistance of primary CML cells to imatinib^66,67^. It should be noted that, a high ROS level not only induces cell apoptosis but also induces noncaspase-dependent necroptosis, which is conducive to overcoming the drug resistance mediated by the apoptotic defect^68,69^.

However, the leukemia niche can protect leukemic cells under oxidative stress. For example, BMSCs protect leukemic cells by activating prosurvival signaling pathways such as the PI3-K/Akt pathway^70^ and releasing protective molecules such as asparagine^71^, fatty acids^72^, and cysteine^73^. Leukemic cells can also relieve oxidative injury by interacting with the leukemia niche. Ding et al.^74^ reported that H_2_O_2_ generated by chronic lymphocytic leukemia (CLL) cells under vorinostat treatment was transferred to the surrounding stromal cells and drove autophagy, mitophagy, and glycolysis, resulting in the local production of high-energy mitochondrial fuels, which were then taken up by CLL cells to be effectively utilized through mitochondrial oxidative phosphorylation to enable more ATP production (Fig. 2). Under daunorubicin treatment, acute lymphoblastic leukemia (ALL) cells induce intracellular ROS production and oxidative stress responses in adipocytes, leading to the secretion of soluble factors that protect ALL cells from daunorubicin^75^.

Leukemic cells also have complex antioxidative mechanisms to resist oxidative stress. The intracellular antioxidant enzyme system consisting of superoxide dismutase (SOD), peroxidase, and CAT can clear away excessive ROS in cells, thus maintaining intracellular redox balance. Some small molecular substances, such as vitamins E, C, and A, can also clear away free radicals and prevent lipid peroxidation. In addition, the mercapto reductive buffer system consisting of glutathione (GSH) and thioredoxin (Trx) has a very important role in maintaining the intracellular redox state^76^. Several studies have demonstrated that suppression of the intracellular antioxidant system can help improve the efficacy of pro-oxidative therapy in leukemia^77–81^. It has been recently reported that nuclear factor erythroid 2-related factor 2 (Nrf2) is a key factor regulating the oxidative stress response of cells. Nrf2 is regulated by Kelch-like ECH-associated protein 1 (Keap1) and participates in the regulation of the activities of antioxidant enzymes such as SOD, CAT, and Trx by interacting with antioxidant response elements (AREs)^82,83^; Nrf2 can also promote the expression of various antioxidant proteins such as heme oxygenase enzyme-1 (HO-1) and regulate the regeneration of GSH^82,83^. Suppressing the Nrf2 signaling pathway has been proven to effectively reverse the drug resistance of leukemia cells^84^. Besides, there are still many antioxidant molecules are involved in protecting leukemia cells from oxidative stress. Under the higher oxidative stress level caused by chemotherapy, ALL cells can upregulate antioxidant production and myeloid cell leukemia 1 expression^85^. Under cytarabine treatment, myeloperoxidase (MPO) expression is increased in AML cells, which promotes the conversion of hydrogen peroxide into hypochlorous acid, thereby reducing the sensitivity of AML cells to cytarabine^86^. In addition, a complex signaling network involving HO-1^87^, AMP-activated protein kinase^88^, STAT5^89^, and STAT3^90^ also has an important role in maintaining redox homeostasis in leukemic cells (Fig. 2).

Recently, many studies have demonstrated that nitric oxide (NO), the main member of ROS, also exhibits antioxidation properties, which can destroy cascade reaction of lipid peroxidation and protect integrity of cytoplasm, avoiding oxidative injury of leukemic cells^91,92^. However, NO also stimulates ROS production, thereby eliciting lipid peroxidation. Therefore, NO has dual roles in the body, which are dependent on its relative concentration in the body^93,94^. Moreover, much evidence shows that leukemic cells relieve oxidative stress via autophagy and clear the organelles damaged by oxidative stress. Thus, inhibiting autophagy is conducive to promoting the death of leukemic cells^95–97^.

Given the facts above, pro-oxidative treatment for leukemia requires an individualized strategy, and specific interventions should be based on the antioxidative features of different types of leukemic cells. Furthermore, blocking the microenvironment’s protection of leukemic cells from antioxidants may contribute to better outcomes.

### Oxidative injury of HSCs caused by pro-oxidative treatment

**Fig. 3:**
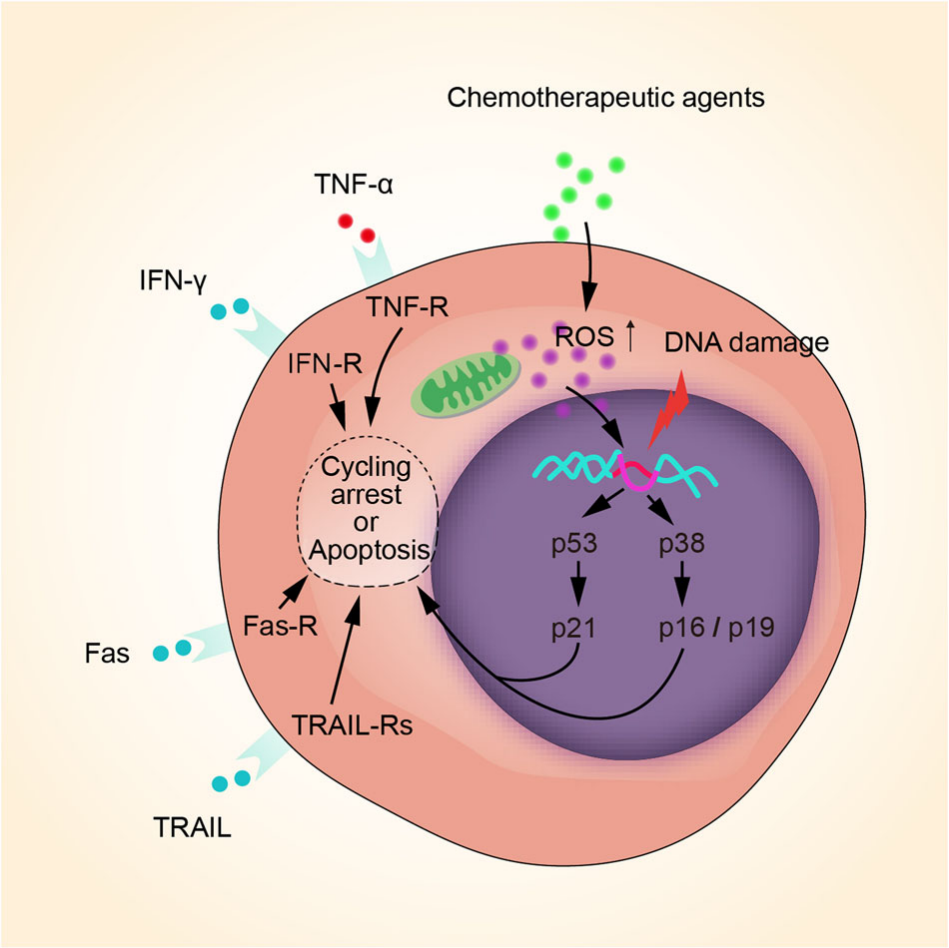
Proposed mechanism of HSCs oxidative damage and senescence. Chemotherapy, especially pro-oxidative chemotherapy, leads to a considerable increase of ROS derived from mitochondria and other resources, inevitably inducing DNA oxidative injury of HSCs. In that case, cell cycle arrest is elicited by p53–p21and p38–p16/19 pathways to repair DNA, whereas cell cycle arrest is the main cause of cell senescence. If DNA injury cannot be repaired, both intrinsic and extrinsic apoptotic pathways will be activated via various pathways, leading to cell apoptosis. Besides, TNF-α, IFN-γ, Fas, and TRAIL bind to their receptors and lead to HSCs cycle arrest or apoptosis.

The killing power of ROS is not cell specific. Therefore, a high ROS level not only kills the leukemic cells but also undesirably causes oxidative injury or even death of HSCs. Tang et al. showed that the high ROS level induced by pro-oxidative chemotherapeutic agents not only caused direct injury and apoptosis of HSCs but also disrupted the BM niche where HSCs reside. This will ultimately damage the hematopoietic functions of the BM^98^. Continuous oxidative injury of DNA can also cause senescence and loss of the self-renewal ability of HSCs, which may be an important reason for long-term BM suppression and hematopoietic failure^99,100^. The proposed mechanism of HSC oxidative damage and senescence is illustrated in Fig. 3.

In addition, recent studies have demonstrated that autophagy serves as an important buffer system for oxidative stress and has a vital role in protecting HSCs from oxidative injury and maintaining the stem cell features of HSCs^101,102^. However, autophagy is also a double-edged sword, and excessive autophagy will cause autophagic cell death^13^. As it has been shown that ROS are an important factor inducing autophagy^103^, the autophagy level of LSCs, which depends on mitochondrial respiration, may be theoretically higher than that of HSCs, which depends on glycolysis^104,105^. However, this hypothesis remains to be further confirmed by more evidence. To date, targeting autophagy to eradicate LSCs via autophagic cell death without disturbing normal hematopoietic cells has aroused widespread attention in researchers^106,107^.

To address the adverse impact of pro-oxidative treatment on BM hematopoietic function, inhibition of leukemic cells’ intracellular antioxidants, e.g., GSH^108^ and HO-1^109^, may be an effective strategy. In theory, antioxidant inhibitors can also cause increased intracellular ROS levels, which means they can be used as pro-oxidants. Isothiocyanates is such a pro-oxidant drug^110^. It was reported that, Isothiocyanates act by depleting GSH pools, and efficiently kill fludarabine-resistant CLL cells^111^ and imatinib-resistant CML cells^112^ selectively without attacking normal hematopoietic cells. Preclinical studies have shown that different SOD inhibitors, such as ATN-224^113^ and 2-methoxyestradiol (2-ME)^114^, have an antileukemia effect. It has been reported that 2-ME can target and kill leukemic cells but has no such effect on normal HSCs^115^.

**Fig. 4:**
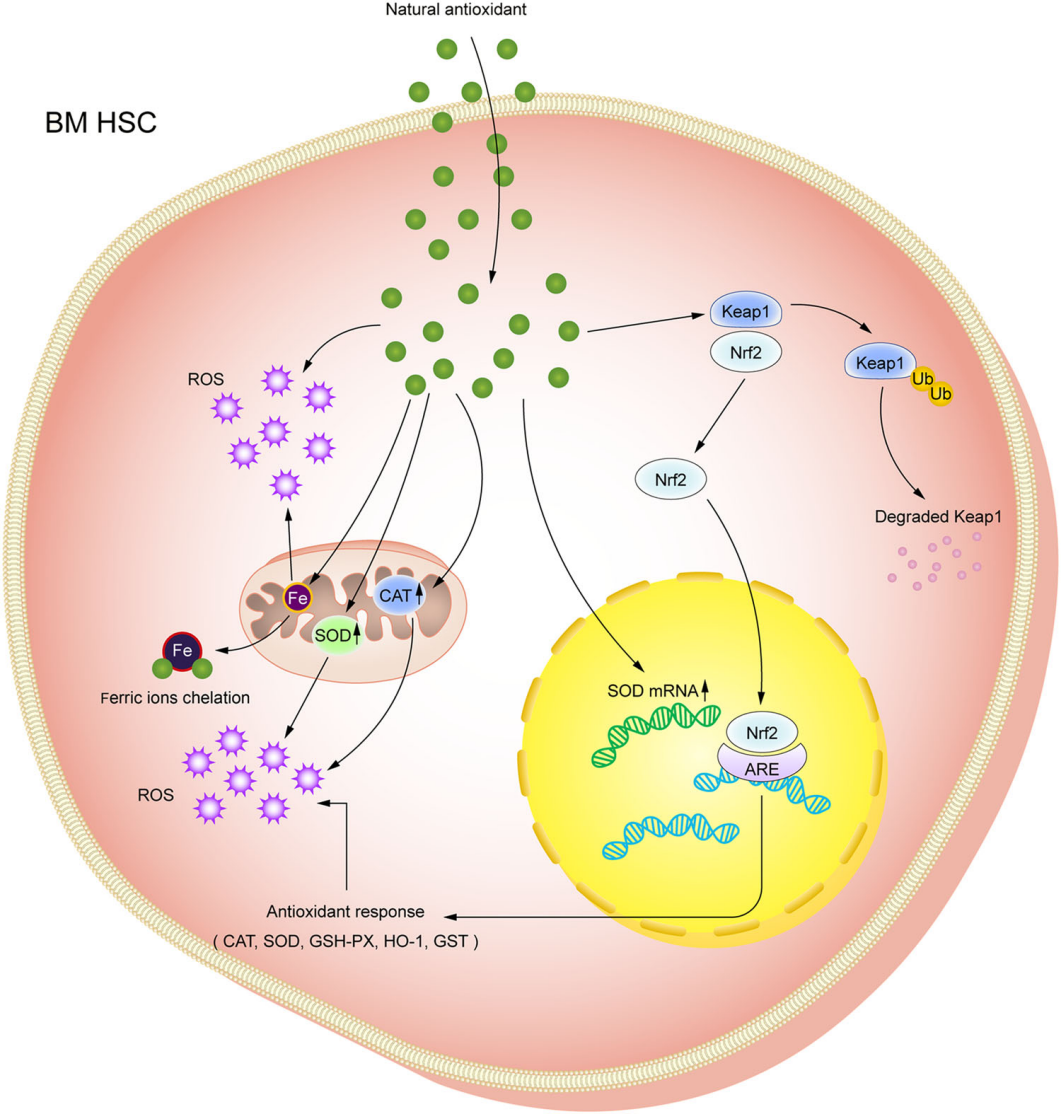
Antioxidative mechanism of natural antioxidants. Natural antioxidants extracted from plants exert antioxidative effects mainly through the following pathways: 1. direct clearing of ROS; 2. chelating ferric ions to inhibit ROS production; 3. improving the activities of anti-oxidases such as SOD and CAT; 4. promoting SOD mRNA expressions; 5. clearing ROS by activating the Nrf2 antioxidative pathway.

In order to relieve the oxidative injury of conventional chemotherapeutic agents on BM hematopoietic tissues, research is underway to screen and develop natural components and biological products with antitumor effects, such as catechins, parthenolide, curcumin, and resveratrol^116–135^. The role of these natural antitumor drugs in inducing the apoptosis of tumor cells is also related to ROS. They can replace conventional chemotherapeutic drugs or reduce the dosage of conventional chemotherapeutic agents to a certain degree, thereby alleviating toxicity and side effects. Zhang et al. showed that catechins suppressed the proliferation of acute promyelocytic leukemia (APL) cells and elicited cell apoptosis at the micromolar concentration level, which was related to mitochondrial damage, ROS production, and caspase activation. Catechin-mediated apoptosis was also found in primary APL cells, but normal hematopoietic progenitor cells were unaffected^121^. According to the report by Guzman et al., parthenolide could induce the apoptosis of primary human AML cells and blast crisis CML cells by inducing ROS generation. However, parthenolide of the same concentration barely had any effects on the BMHCs^130^. The above studies suggest that natural antitumor drugs have broad prospects in the treatment of leukemia while protecting BM hematopoietic function. Some natural compounds that exert antileukemia activity via ROS-dependent actions are listed in Table 1. In addition, recent studies have found that many natural compounds possess potent antioxidant activity, which may protect BMHSCs from oxidative damage. It has been reported that some natural polyphenolic antioxidants, such as curcumin and quercetin, can effectively protect BMHSCs from the oxidative damage caused by pro-oxidative drugs without affecting the antileukemia function^136–138^. It has been shown that this phenomenon is associated with the difference in ROS levels between cancer cells and healthy cells, and polyphenols may exert more pro-oxidative action in cancer cells with increased levels of ROS^125,138^. In recent years, many studies have shown that many alkaloids, polysaccharides, flavonoids, and saponins extracted from plants also display antitumor and antioxidant effects^139–141^. The antioxidative mechanism of natural antioxidants is illustrated in Fig. 4.

**Table 1:** Natural compounds that exert antileukemia effects via ROS-dependent actions.

Natural compound	Cell type	Action	Possible antileukemia mechanism	Ref.
Alkanone-gingerol	Human CML cell lines K562, LAMA-84, JURL-MK1; Human AML cell lines U937, HL-60, NB4; Primary cells isolated from peripheral blood (PB) of patients with myeloid leukemia and normal healthy donors	Inducing myeloid leukemia cell death, while having little cytotoxicity on the normal peripheral blood mononuclear cells (PBMCs)	Initiated by ROS and mediated through an increase in miR-27b expression and DNA damage	^116^
Ardisiacrispin B	Human T-cell ALL cell line CCRF-CEM	Inducing apoptosis	Activating caspases 8 and 9 and caspase 3/7 and increasing ROS production	^117^
Artesunate	Human T-cell ALL cell lines Jurkat, CEM, and Molt-4	Inducing apoptosis	ROS-dependent mitochondria-mediated pathway	^118^
Avocatin B	Human AML cell lines OCI-AML2, TEX, and primary AML cells isolated from the PB of AML patients	Reducing human primary AML cell viability without effects on normal HSCs, and inducing apoptosis in AML cells	ROS-dependent mitochondria-mediated pathway	^119^
Baicalin	Human T-cell ALL cell line Jurkat, human PBMCs isolated from blood of healthy volunteers	Inducing apoptosis in Jurkat cells, while having little cytotoxicity on PBMCs	ROS-dependent mitochondria-mediated pathway	^120^
Catechin	Human acute promyelocytic leukemia (APL) cell lines NB4, NB4-R1 and NB4-R2; Human AML cell lines Kasumi-1, K562 and U937; Primary leukemia cells isolated from the bone marrow of APL patients	Inhibiting APL cell proliferation and inducing apoptosis	Inducing APL cell apoptosis through intrinsic apoptotic pathway via Bcl-xL downregulation and ROS induction.	^121^
Cathachunine	Human acute promyelocytic leukemia cell line HL-60, and CML cell line K562	Inducing apoptosis in human HL-60 and K562 leukemia cells	ROS-dependent mitochondria-mediated intrinsic pathway, and is regulated by the Bcl-2 protein family.	^122^
Cinnamaldehyde	Human acute promyelocytic leukemia cell line HL-60	Inducing apoptosis	ROS-dependent mitochondria-mediated pathway	^123^
Curcumin	Human B-cell precursor Leukemia cell Lines 697, REH, RS4;11, and SupB15	Inducing apoptosis	ROS-dependent mitochondria-mediated intrinsic pathway	^124^
Cyanidin-3-rutinoside	Human promyelocytic leukemia cell line HL-60, human T-cell ALL cell lines CCRF-CEM and Molt-4, etc; Human PBMCs isolated from healthy donors	Inducing apoptosis in leukemia cell lines, while having little cytotoxicity on the normal PBMCs	ROS-dependent activation of p38 MAPK and JNK	^125^
Dioscin	Human acute promyelocytic leukemia cell line HL-60	Inducing apoptosis	ROS-dependent mitochondria-mediated pathway	^126^
Emodin	Murine myelomonocytic leukemia cell line WEHI-3	Inducing apoptosis	Endoplasmic reticulum (ER) stress, cascade-dependent and -independent mitochondrial pathways	^127^
Hydroxychavicol	Human CML cell line K562	Sensitizing imatinib-resistant CML cells to TRAIL-induced apoptosis	Downregulating the antiapoptotic proteins XIAP and FLIP via ROS-dependent actions	^128^
Medicarpin	Human CML cell lines K562, LAMA-84; Human AML cell lines U937, OCIAML-3; PBMCs isolated from normal healthy donors and AML patients	Sensitizing myeloid leukemia cells to TRAIL-induced apoptosis, while having little cytotoxicity for primary normal PBMCs.	Upregulating of DR5 through activation of the ROS-JNK-CHOP pathway	^129^
Parthenolide	AML cells, blast crisis CML (bcCML) cells, normal BM cells, and umbilical cord blood cells obtained from volunteer donors	Inducing robust apoptosis in primary human AML cells and bcCML cells without affecting normal stem and progenitor cells	Inhibiting NF-kappaB, activating p53, and increasing ROS	^130^
Platycodon D	Human acute monocytic leukemia cell line U937	Inducing apoptosis	ROS-dependent mitochondria-mediated pathway	^131^
Quercetin	Human CML cell line CCL-243	Inducing apoptosis and inhibiting growth	ROS-dependent mitochondria-mediated pathway	^132^
Resveratrol	Human AML cell lines U937 and MV-4-11, and primary AML cells isolated from BM or PB of AML patients	Sensitizing AML cells to histone deacetylase inhibitors	Multiple ROS-dependent actions including death receptor upregulation, extrinsic apoptotic pathway activation, and DNA damage induction.	^133^
Taxol	Human CML cell line K562	Inducing apoptosis	Inducing intracellular oxidative stress and JNK pathway activation	^134^
Triptolide	Human AML cell line KG1a	Inducing apoptosis	Inducing ROS generation and inhibiting the Nrf2 and HIF-1α pathways	^135^

The researches listed in the table are divided into 3 sections according to the cells studied, primary cells (Ref. ^130^); cell lines (Refs. ^117,118,122–124,122–124,126–128,131,132,134,135^); primary cells and cell lines (Refs. ^116,119–121,125,129,133^).

### Summary

New treatment strategies targeting the oxidative stress status of leukemic cells and the BM microenvironment have become hot topics of research. However, many of the previous studies have limitations. First, most of them are in vitro studies, which cannot completely simulate the BM microenvironment in a physical or pathological condition. Therefore, these studies cannot truly reflect the state inside the leukemia patients’ bodies. Second, leukemia is highly heterogeneous and varies across individuals, disease types, and development stages. Moreover, the cell status, oxidative stress level of cells and relevant regulation mechanisms also vary, making it very difficult to determine the appropriate ROS level for pro-oxidative treatments. Third, there are also BMSCs and HSCs in the BM microenvironment apart from the leukemic cells. Pro-oxidant drugs may damage HSCs and other normal cells, leading to severe BM suppression and other adverse events. In conclusion, deeper and more comprehensive studies of the redox features and regulatory mechanisms of HSCs and LSCs in the BM microenvironment are urgently needed to identify new therapeutic targets to eradicate LSCs without harming normal HSCs. This issue represents the biggest challenge in the field of leukemia treatment.

In recent years, many studies have made good use of modern biological technology. Great efforts have been devoted to developing animal models that can realistically reflect the microenvironment of leukemia patients, including transgenic models and chimeric models. The findings from these models are encouraging further studies on leukemia. With the establishment of more animal models of leukemia with high fitness, our understanding of leukemia will deepen, which will contribute to progress in leukemia studies.
